# Economic burden of asthma multimorbidity in Singapore: Shadow costs of steroid use^☆^

**DOI:** 10.1016/j.waojou.2025.101146

**Published:** 2025-11-27

**Authors:** Laura Huey Mien Lim, Yah Ru Juang, Sanjay H. Chotirmall, Kelvin Bryan Tan, Mariko Siyue Koh, John A. Abisheganaden, David B. Price, Ming-Ju Tsai, Mei Fong Liew, Pei Yee Tiew, Anthony Chau Ang Yii, Wenjia Chen

**Affiliations:** a Saw Swee Hock School of Public Health, National University of Singapore, Singapore, Singapore; b Lee Kong Chian School of Medicine, Nanyang Technological University, Singapore, Singapore; c Department of Respiratory and Critical Care Medicine, Tan Tock Seng Hospital, Singapore; d Ministry of Health, Singapore, Singapore; e Department of Respiratory and Critical Care Medicine, Singapore General Hospital, Singapore, Singapore; f Duke-NUS Medical School, National University of Singapore, Singapore, Singapore; g Health Services and Outcomes Research, National Healthcare Group, Singapore; h Observational and Pragmatic Research Institute, Singapore, Singapore; i Optimum Patient Care, Cambridge, UK; j Centre of Academic Primary Care, Division of Applied Health Sciences, University of Aberdeen, Aberdeen, UK; k Division of Pulmonary and Critical Care Medicine, Department of Internal Medicine, Kaohsiung Medical University Hospital, Kaohsiung Medical University, Kaohsiung, Taiwan; l Department of Internal Medicine, School of Medicine, College of Medicine, Kaohsiung Medical University, Kaohsiung, Taiwan; m Division of Respiratory and Critical Care Medicine, Department of Medicine, National University Hospital, National University Health System, Singapore, Singapore; n Department of Medicine, Yong Loo Lin School of Medicine, National University of Singapore, Singapore, Singapore; o FAST and Chronic Programmes, Alexandra Hospital, National University Health System, Singapore, Singapore; p Department of Respiratory and Critical Care Medicine, Changi General Hospital, Singapore, Singapore

**Keywords:** Asthma, Comorbidity, Oral corticosteroid, Economic burden, Direct cost

## Abstract

**Background:**

In some countries including Singapore, biologic therapies are not routinely available. Instead, oral corticosteroid (OCS) is commonly used for severe asthma management, which could lead to substantial adverse health events.

**Objective:**

To estimate the multimorbidity costs in asthma patients from a multi-ethnic Asian population.

**Methods:**

We examined national health administrative data (2012–2019) from Singapore. Direct medical costs were summed from costs of hospitalisation, emergency department (ED), specialist care, and public primary care. Prescription data were not available but formed part of public primary care costs. We measured cost per patient-year (PY) in 2023 Singaporean dollars (SGD$1 = US$0.76 = ₤0.60 = €0.69). We performed propensity-score matching on asthma and non-asthma patients, and applied generalised linear models to estimate total and excess costs due to asthma, OCS-related comorbidities, and other comorbidity groups.

**Results:**

We identified 19,979 paediatric and 48,237 adult asthma patients (48.2% males, 50.4% Chinese, 13.9% Indian, 26.8% Malay), and matched equal number of non-asthma patients. Paediatric and adult asthma patients respectively incurred $816.3/PY (95% CI: $803.0/PY-$829.5/PY) and $1855.9/PY (95% CI: $1845.0/PY-$1871.0/PY) in total costs. The average ($1610.9/PY [95% CI: $1599.5/PY-$1621.3/PY]) was thrice of non-asthma patients’ ($530.4/PY). Excess costs (mean = $927.2/PY) were driven by asthma ($403.0/PY), OCS-related comorbidities ($104.0/PY), other metabolic disease ($116.4/PY), circulatory diseases ($112.9/PY) and non-asthma respiratory conditions ($107.4/PY). All excess cost components increased steadily over the 8-year study period.

**Conclusion:**

The burden of asthma multimorbidity in Singapore is severe, with a considerable fraction attributable to OCS-related comorbidities. Policies should aim to reduce excess OCS use and enhance integrated multimorbidity management.

## Introduction

Asthma is a complex and heterogenous airway disease affecting 262 million people worldwide,^1^ with an estimated 100 million new cases expected globally in the next decade.^2^ Due to common metabolic pathways and inflammatory alternations including systemic inflammation and inflammasome, asthma is associated with significant comorbidities which include but are not limited to respiratory, cardiovascular, metabolic, and psychiatric conditions.^3^ Based on data from a large Caucasian population in Canada, comorbidity-attributable excess medical cost was 5 times as high as asthma-attributable costs, with the costliest components found to be psychiatric, digestive, and nervous disorders.^4^ In severe asthma patients (5%–10% of the asthma population),^5^ non-asthma respiratory conditions form the largest component of comorbidity costs, which make up half of total excess costs relative to non-severe asthma.^6^

Globally, severe asthma is commonly treated with oral corticosteroid (OCS) to alleviate airway inflammation during acute exacerbations. However, OCS use even in low doses is associated with significant adverse health events, leading to substantial incremental costs.^7^^,^^8^ These costs are sometimes referred to as “shadow costs” of OCS, as they are often eluded by the low acquisition costs of OCS, but they should not be overlooked.^8^^,^^9^ In Asia, OCS is commonly used not only in severe asthmatics but also amongst some with mild-to-moderate asthma.^10^ Coupled with the added influence of Asia-unique multimorbidity clusters including cardiorespiratory and cardiopulmonary-mental-degenerative diseases,^11^ the resulting burden associated with OCS use is potentially high. However, Asian countries face a lack of comprehensive long-term claims data to study the patterns of ongoing OCS use and its additional costs in asthma patients.^12^

In Singapore, asthma affects 8.9% of children^13^ and 5.1% of adults.^14^ With its multi-ethnic makeup (74% Chinese, 14% Malay, 9% Indian, 3% other), the Singapore asthma population is characterised by diverse phenotypic profiles and comorbidity risks across ethnic groups.^15^^,^^16^ The total annual economic burden of asthma is a staggering SGD$2.09 billion (USD$1.50 billion),^17^ possibly owing to gaps in asthma care including a lack of asthma follow-up, low rates of controller use,^18^ and high asthma acute care burdens.^19^ Cost barriers also exist, as only 6% of asthma patients receive full reimbursement whereas over 64% received partial reimbursement in Singapore.^20^ Meanwhile, approximately 15% of local asthma patients receive prednisolone,^21^ and those with severe asthma receive an average of 2.6 OCS courses per year.^12^ The high use of OCS reflects challenges in asthma management in Asia, which are likely related to reactive, event-driven care and suboptimal treatment goals.^10^ Moreover, severe asthma patients typically lack access to biologic therapies.^10^ Despite the substantial burden, till date, population-based economic evidence on asthma-related burdens remains limited in Singapore. More importantly, the costs of asthma comorbidities had not been investigated before.

In line with Singapore's ongoing healthcare reform “Healthier SG”^22^ which aims to strengthen preventive care and optimise value-based disease management, the recent launch of a nationwide health administrative dataset in Singapore provides a timely opportunity to address current evidence gaps. Using eight-year health administrative data from Singapore, this study aimed to estimate the direct medical costs of asthma and comorbidities, particularly OCS-related comorbidities, in Asian asthma patients.

## Materials and methods

### Data

We analysed linked individual-level health administrative data on the Trusted Research and Real Word Data Utilisation (TRUST) platform. The data comprised sociodemographic and claims records of 4.1 million Singaporean residents. Based on the period of available data of the respective healthcare settings in TRUST, we retrieved and merged episode-level data on hospitalisations from 1998 to 2020, emergency department (ED) and specialist care visits from 2006 to 2020, and primary care visits from 2012 to 2020. Data on hospitalisation, ED visits and specialist care encompassed records from >90% of public and private hospitals in Singapore, whereas primary care data only included those from the public healthcare sector. Missing data (<10%) in baseline sociodemographic variables were imputed using a non-parametric random forest-based imputation algorithm.

### Study design and sample

This was a retrospective, propensity-score matched cohort study. A schematic diagram of the study design was presented in Supplemental Fig. 1.

We created an asthma cohort by identifying patients, of all ages, who fulfilled a validated case definition of asthma, ie, having at least 1 inpatient encounter or at least 2 outpatient encounters on different dates, where asthma was the final diagnosis, during any 12-month period (sensitivity = 0.632, specificity = 0.997).^23^ Asthma-specific healthcare encounters were determined based on International Classification of Diseases (ICD) codes (ICD-9: 493.x except 493.2; ICD10: J45.x, J46.x). The index date was defined as the date of the first recorded asthma-related healthcare encounter. Patients with a diagnosis of chronic obstructive pulmonary disease (COPD, ICD-9: 491.x, 492.x, 496.x; ICD-10: J43.x, J44.x) within 2 years around the index date, were excluded.

Subsequently, we created a comparison group consisting of patients of all ages (age 0 onwards) with no asthma-specific resource use but had at least 1 recorded healthcare encounter during the study period. The index date of the comparison subjects was defined as the date of their first recorded healthcare encounter after the initial 12 months of available records. In both cohorts, patients must have at least 1 year of pre-index washout period and at least 1 year of follow-up data from the index date.

We performed propensity score matching to create a balanced 1:1 cohort of asthma and non-asthma patients. Propensity score matching mitigates selection bias and improves unbiased effect estimation by balancing exposure-related covariates between the exposed and comparison groups, emulating a controlled trial design.^24^ The cohorts were matched on baseline age, sex, ethnicity, socioeconomic status (SES), residency, and calendar year of the index date when asthma was first identified. We performed matching based on nearest-neighbourhood matching without replacement.

We analysed data in the common, available data period, ie, 2012–2019. All subjects were followed from their index date or January 1, 2012, whichever came first, to their date of death or December 31, 2019, whichever came first. Data in 2020 was omitted to exclude the temporal effects of the COVID-19 pandemic on patterns of healthcare utilisation.

### Outcomes

Cost outcomes were adjusted to 2023 Singapore dollars (SGD$1 = US$0.76 = ₤0.60 = €0.69) based on consumer price indices of the healthcare sector.^25^ Direct medical costs were summed from hospitalisation, ED and outpatient costs, where the latter consisted of primary care visit and specialist care costs (medication costs included). Hospitalisation and primary care visit costs were directly extracted from episode-level billing records. Specialist care episodes were identified from hospitalisation records by corresponding codes and same-day discharge. ED episodes were partially captured (8.4%) in the hospitalisation dataset (identified by the joint criteria of same-day discharge, absence of one-day procedure and specialist care), while the majority (91.6%) were covered in the ED dataset.

All-cause medical costs were broken down into asthma- or comorbidity-attributable costs, respectively derived by attributing healthcare encounters to a primary diagnosis of asthma or major comorbidity categories. Comorbidity-attributable costs were derived by characterising episode-level healthcare costs by diagnosis groups including OCS-related comorbidities, non-respiratory major comorbidity categories, non-asthma respiratory conditions, and all other comorbidities. OCS-related comorbidities included a set of adverse health events which are commonly associated with OCS use. Consistent with well-established comorbidity categories shown to be associated with OCS adverse effects,^26^, ^27^, ^28^ we classified the following conditions under OCS-related comorbidities: heart failure, myocardial infarction, osteoporosis, stroke, glaucoma, cataract, pulmonary embolism, depression, anxiety, type 2 diabetes, renal failure, peptic ulcer, pneumonia, and obstructive sleep apnoea. This categorisation is widely applied in studies examining OCS use and its impacts in asthma patients globally.^26^^,^^29^, ^30^, ^31^, ^32^ Supplemental Tables 1 and 2 detail the diagnosis codes of OCS-related comorbidities and other major comorbidity categories. Healthcare records with a missing diagnosis or cost value were excluded.

### Statistical analysis

All analyses were performed using R version 4.1.1. The unit of analysis was patient-year (PY). Outlying values of per-PY costs were truncated on the 97.5th percentile. For each cohort, we estimated component-specific (hospitalisation, ED, primary care, specialist care, total) and disease-specific (asthma-attributable, comorbidity-attributable, all-cause) costs using separate generalised linear models with the normal distribution and identity link, in line with best practices in large sample sizes.^33^ To derive excess costs, which were estimated as the adjusted differences in the predicted costs between an asthma patient and the non-asthma matched patient, we applied a robust causal inference technique, generalised computation or G-computation.^34^ G-computation is a robust causal inference method that estimates covariate-adjusted effects of an exposure, which is implemented by predicting and contrasting outcomes within each individual under counterfactual exposure scenarios, and then averaging out the exposure effect across the sample; in this process, the bootstrapping method is often used to obtain robust inference estimates. This approach serves to decouple confounding adjustment and effect estimation.^34^ We further estimated marginal effects of covariates on excess costs, defined as the change in the expected excess costs due to a change in the level of exposure, keeping other covariates constant. Robust inference was derived through repeated bootstrapping.

Independent variables included age-sex subgroup (males and females respectively aged 0–18, 19–64, or 65 years and above), ethnicity (Chinese, Indian, Malay, Others), SES (high, middle, low) and residency status (resident, non-resident). SES was determined by housing type based on a modified Singapore Housing Index (SHI).^35^ The models also included calendar year and follow-up year.

To characterise high-cost asthma patients, we estimated excess costs of patient-years with the top 10% highest OCS-related comorbidity-attributable costs, and patient-years with 2 or more asthma-related hospitalisations or ED visits. We modelled Least Absolute Shrinkage and Selection Operator (LASSO) regression to select essential predictors of high-cost patients, including baseline sociodemographic characteristics and a 12-month history of comorbidities.

This study complied with the Declaration of Helsinki. Ethics approval for exempted review was obtained from the Institutional Review Board (IRB) of National University of Singapore (NUS-IRB-2021-967).

## Results

Table 1 compares the baseline characteristics and healthcare utilisation of our study sample (patient selection flowcharts presented in Supplemental Fig. 2). We analysed 68,216 asthma patients consisting of 19,979 paediatric (29.3%) and 48,237 adult patients (70.7%), and 68,216 matched non-asthma patients. In the asthma cohort, 48.2% were males, and the ethnic composition was 50.4% Chinese, 13.8% Indian, 26.8% Malay and 9% of other races.

**Table 1:** Baseline characteristics of matched patient sample

Characteristics	ASTHMA (N = 68,216)			NON-ASTHMA (N = 68,216)			SMD
	Overall (N = 68,216)	Paediatric (0–18 years) (N = 19,979)	Adult (>18 years) (N = 48,237)	Overall N = 68,216)	Paediatric (0–18 years) (N = 18,853)	Adult (>18 years) (N = 49,363)	Overall
Age, mean (SD)	31.8 (21.9)	6.7 (6.2)	42.2 (17.1)	33.4 (22.7)	5.2 (5.9)	44.2 (16.7)	0.072
Age group, n (%)							0.035
0-18	19,979 (29.3)	19,979 (100)	0 (0)	18,853 (27.6)	18,853 (100)	0 (0)	
19-64	42,538 (62.4)	0 (0)	42,538 (88.2)	43,027 (63.1)	0 (0)	43,027 (87.2)	
65 or above	5699 (8.4)	0 (0)	5699 (11.8)	6336 (9.3)	0 (0)	6336 (12.8)	
Gender, n (%)							0.003
Male	32,907 (48.2)	11,936 (59.7)	20,971 (43.5)	33,007 (48.4)	9703 (51.5)	23,304 (47.2)	
Female	35,309 (51.8)	8043 (40.3)	27,266 (56.5)	35,209 (51.6)	9150 (48.5)	26,059 (52.8)	
Ethnicity							0.069
Chinese	34,365 (50.4)	9661 (48.4)	24,704 (51.2)	32,275 (47.3)	9002 (47.7)	23,273 (47.1)	
Indian	9446 (13.8)	2272 (11.4)	7174 (14.9)	10,152 (14.9)	2338 (12.4)	7814 (15.8)	
Malay	18,285 (26.8)	6604 (34.1)	11,681 (24.2)	18,757 (27.5)	5908 (31.3)	12,849 (26)	
Others	6120 (9)	1442 (7.2)	4678 (9.7)	7032 (10.3)	1605 (8.5)	5427 (11)	
SES, n (%)							0.026
Low	22,461 (32.9)	6685 (33.6)	15,776 (32.7)	22.351 (32.8)	6244 (33.1)	16,107 (32.6)	
Middle	36,977 (54.2)	10,823 (54.2)	26,154 (54.2)	36,500 (53.5)	10,091 (53.5)	26,409 (53.5)	
High	8778 (12.9)	2471 (12.4)	6307 (13.1)	9365 (13.7)	2518 (13.4)	6847 (13.9)	
Residency status, n (%)							0.035
Resident	64,003 (93.8)	18,816 (94.2)	45,187 (93.7)	63,411 (93)	17,627 (93.5)	45,784 (92.7)	
Non-resident	4213 (6.2)	1163 (5.8)	3050 (6.3)	4805 (7)	1226 (6.5)	3579 (7.2)	
Baseline comorbidities, n (%)							
OCS-Related comorbidities	10,167 (14.9)	910 (4.6)	9252 (19.3)	7193 (10.5)	850 (4.5)	6343 (12.9)	0.131
Heart failure	675 (1)	8 (0)	666 (1.4)	377 (0.6)	3 (0)	374 (0.8)	0.050
Myocardial infection	423 (0.6)	6 (0)	416 (0.9)	599 (0.9)	0 (0)	599 (1.2)	0.030
Osteoporosis	690 (1)	4 (0)	686 (1.4)	43 (0.1)	2 (0)	41 (0.1)	0.130
Stroke	1269 (1.9)	14 (0.1)	1254 (2.6)	1039 (1.5)	7 (0)	1032 (2.1)	0.026
Pulmonary embolism	29 (0)	0 (0)	29 (0.1)	33 (0)	0 (0)	33 (0.1)	0.003
Glaucoma	348 (0.5)	5 (0)	343 (0.7)	185 (0.3)	3 (0)	182 (0.4)	0.038
Cataract	2796 (4.1)	20 (0.1)	2775 (5.8)	1608 (2.4)	4 (0)	1604 (3.2)	0.099
Renal failure	1189 (1.7)	4 (0)	1185 (2.5)	305 (0.4)	2 (0)	303 (0.6)	0.125
Type 2 diabetes	6008 (8.8)	84 (0.4)	5921 (12.3)	1879 (2.8)	25 (0.1)	1854 (3.8)	0.262
Peptic ulcer	90 (0.1)	6 (0)	84 (0.2)	15 (0)	1 (0)	14 (0)	0.040
Pneumonia	2563 (3.8)	788 (3.9)	1774 (3.7)	1542 (2.3)	803 (4.3)	739 (1.5)	0.088
Obstructive sleep apnoea	0 (0)	0 (0)	0 (0)	0 (0)	0 (0)	0 (0)	<0.001
Major comorbidity groups							
Circulatory	11,046 (16.2)	366 (1.8)	10,670 (22.2)	4824 (7.1)	176 (0.9)	4647 (9.4)	0.287
Digestive	12,834 (18.8)	2893 (14.5)	9905 (20.6)	11,198 (16.4)	2491 (13.2)	8707 (17.6)	0.063
Genitourinary	9121 (13.4)	1374 (6.9)	7735 (16.1)	5054 (7.4)	998 (5.3)	4056 (8.2)	0.196
Musculoskeletal	14,267 (20.9)	1981 (9.9)	12,253 (25.5)	5697 (8.4)	607 (3.2)	5090 (10.3)	0.361
Nervous	4379 (6.4)	867 (4.3)	3491 (7.3)	2264 (3.3)	445 (2.4)	1819 (3.7)	0.144
Metabolic	12,448 (118.2)	450 (2.3)	11,982 (25)	3452 (5.1)	95 (0.5)	3357 (6.8)	0.420
Non-asthma respiratory	21,982 (32.2)	5448 (27.3)	16,484 (34.3)	14,858 (21.8)	6623 (35.1)	8233 (16.7)	0.237
Other	23,051 (33.8)	5254 (26.3)	17,748 (37)	37,711 (55.3)	11,660 (61.8)	26,046 (52.8)	0.443
Baseline HCU per PY, mean (SD)							
Hospitalisation	0.7 (3.2)	0.4 (1.9)	0.8 (3.6)	0.5 (0.5)	0.6 (0.5)	0.4 (0.5)	0.089
ED visit	2 (7.1)	1.8 (6.4)	2 (7.4)	0.4 (0.6)	0.4 (0.6)	0.4 (0.6)	0.310
Primary care visit	14 (31.9)	4.8 (14.3)	17.9 (36.2)	0.3 (1.1)	0.1 (0.6)	0.3 (1.2)	0.609
Specialist care visit	0.3 (1.3)	0.1 (0.6)	0.4 (1.5)	0.1 (0.3)	0 (0.1)	0.1 (0.3)	0.247
Baseline hospital bed-days per PY, mean (SD)	2.8 (17.7)	1.2 (8.2)	3.5 (20.3)	1.9 (6.2)	2.1 (5.8)	1.9 (6.5)	0.068

**Abbreviations:** ED: emergency department; HCU: healthcare utilisation; OCS: oral corticosteroid; PY: patient-year; SES: socioeconomic status; SD: standard deviation; SMD: standardised mean difference.

Compared to non-asthma patients, asthma patients had substantially higher baseline prevalence of endocrine, nutritional and metabolic diseases (or simply metabolic diseases), circulatory, musculoskeletal, and non-asthma respiratory conditions, as well as T2 diabetes (standardised mean difference or SMD>0.2). At baseline, the asthma cohort also incurred higher per-PY episodes of all-cause hospitalisations (mean = 0.7, SMD = 0.1), ED visits (mean = 2, SMD = 0.3), primary care visits (mean = 14, SMD = 0.6) and specialist care visits (mean = 0.3, SMD = 0.2). Supplemental Fig. 3 showed a descriptive summary of follow-up healthcare utilisation volumes. Across 2012-2015 and 2016–2019, paediatric and adult asthma patients consistently incurred higher healthcare utilisation per follow-up PY as compared to their non-asthma counterparts.

All costs are reported in 2023 Singaporean dollars (SGD$1 = US$0.76 = ₤0.60 = €0.69). On average, asthma patients incurred $1610.9/PY (95% CI: $1599.5/PY-$1621.3/PY) in all-cause direct medical costs, with a mean of $816.3/PY (95% CI: $803.0/PY-$829.5/PY) in paediatric patients (0–18 years) and $1855.9/PY (95% CI: $1845.0/PY-$1871.0/PY) in adults (>18 years). Of these, 44.6% were primary care costs which included medication costs ($717.8/PY), 31.7% were hospitalisation costs ($510.2/PY), 19.8% were ED visit costs ($319.5/PY) and 3.9% were specialist care costs inclusive of medication costs ($63.4/PY). In non-asthma patients, total costs were much lower, ie, $525.8/PY (95% CI: $519.1/PY- $536.0/PY), with an average $356.6/PY (95% CI: $346.4/PY-$369.3/PY) in paediatric patients and $580.9/PY (95% CI: $569.4/PY-$595.0/PY) in adult patients, mainly driven by hospitalisation costs ($346.5/PY, 65.3%).

**Fig. 1:**
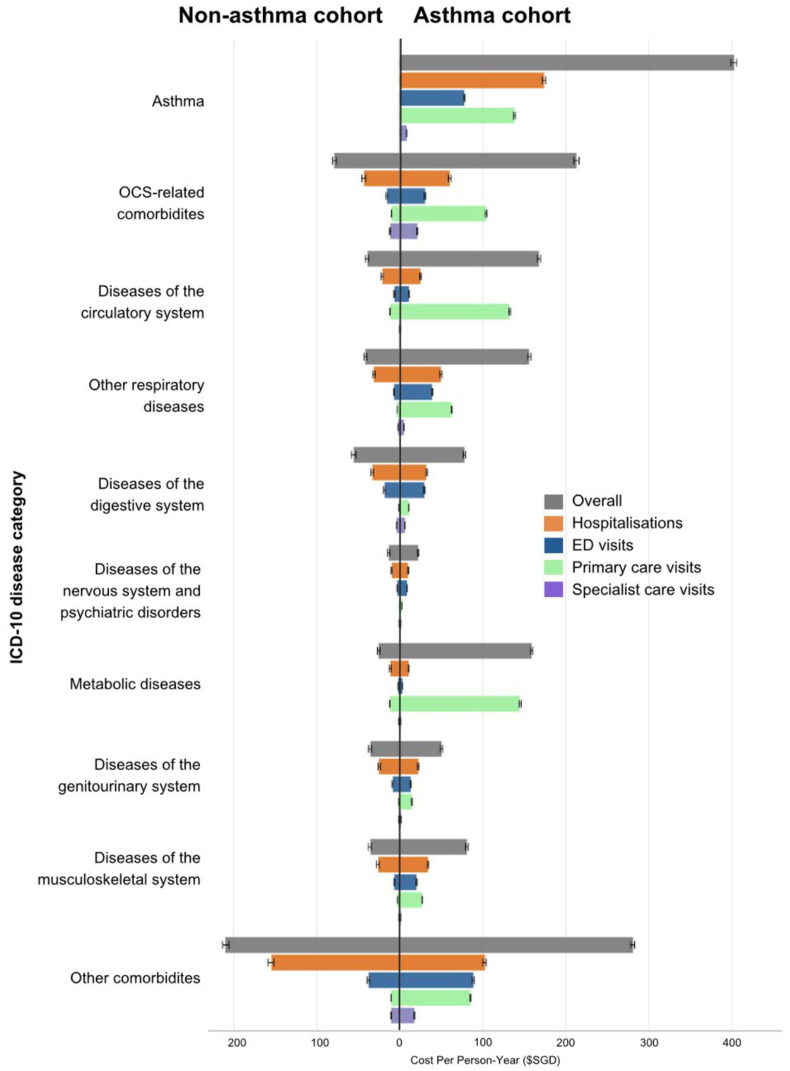
Annualised Total Cost of Multimorbidity in Asthma Patients. Fig. 1 shows the estimated costs in asthma and non-asthma patients by disease category. The error bars represent the 95% confidence intervals (Cis) of the cost estimates. All costs were measured in 2023 Singaporean dollars (SGD$1 = US$0.76 = ₤0.60 = €0.69). Abbreviations: ED: emergency department; ICD: International Classification of Diseases; OCS. oral corticosteroid; SGD: Singaporean dollar

Fig. 1 summarises the estimated costs in asthma and non-asthma patients by disease category (cost estimates detailed in Supplemental Table 3). Costs of asthma patients were mainly attributable to asthma itself ($403.0/PY [95% CI: $400.3/PY-$406.5/PY], 25%), OCS-related comorbidities ($213.0/PY [95% CI: $209.8/PY-$215.9/PY]), other circulatory diseases ($167.5/PY [95% CI: $165.6/PY-$170.0/PY]), metabolic diseases ($158.9/PY [95% CI: $157.6/PY-$160.3/PY]), and non-asthma respiratory conditions ($155.9/PY [95% CI: $154.2/PY-$158.0/PY]), followed by other musculoskeletal diseases ($80.8/PY [95% CI: $79.1/PY-$82.3/PY]), digestive diseases ($77.9/PY [95% CI: $76.1/PY-$79.2/PY]), genitourinary diseases ($50.4/PY [95% CI: $48.9/PY-$51.8/PY]), and nervous diseases ($22.1/PY [95% CI: $21.4/PY-$22.8/PY]), while all other comorbidities accounted for $281.1/PY (95% CI: $278.0/PY-$283.7/PY).

**Fig. 2:**
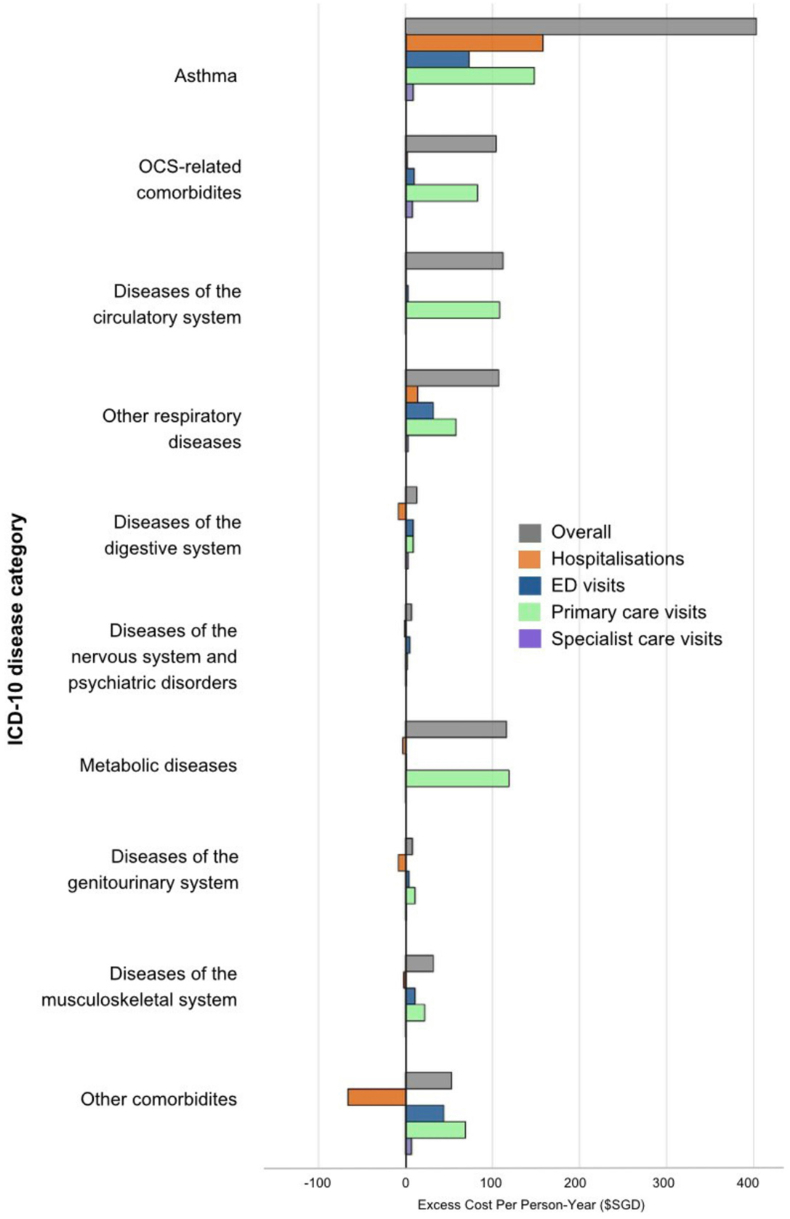
Excess Cost of Multimorbidity in Asthma Patients versus Non-asthma Patients. Fig. 2 shows the excess costs of asthma versus non-asthma patients by disease category. All costs were measured in 2023 Singaporean dollars (SGD$1 = US$0.76 = ₤0.60 = €0.69). Abbreviations: ED: emergency department; ICD: International Classification of Diseases; OCS. oral corticosteroid; SGD: Singaporean dollar

Fig. 2 shows a breakdown of excess costs by disease category (excess cost estimates can be found in Supplemental Table 3). Compared to non-asthma patients, asthma patients incurred an excess $927.2/PY, mainly driven by asthma ($403.0/PY), OCS-related comorbidities ($104.0/PY), other circulatory diseases ($112.9/PY), metabolic diseases ($116.4/PY) and non-asthma respiratory conditions ($107.4/PY). The largest component of excess cost was primary care costs ($613.4/PY, 66.2%), followed by ED visit costs ($197.2/PY, 21.3%), hospitalisation costs ($86.6/PY, 9.4%) and specialist care costs ($30.0/PY, 3.2%). Particularly in OCS-related comorbidities, excess costs were low for hospitalisations, ED visits and specialist care visits; however, primary care visit costs were substantially higher among asthma patients.

Table 2 displays the marginal effects of covariates on per-PY excess costs. Relative to the paediatric group, total excess cost was $613.0/PY (95% CI: $593.6/PY-$632.4/PY) higher in adults (19–64 years), and $1701.5/PY (95% CI: $1669.8/PY-$1733.1/PY) greater in the elderly group (65 year and above). Excess costs were $327.7/PY (95% CI: $$313.4/PY-$342.0/PY) lower in males than in females, and $720.6/PY (95% CI: $678.0/PY-$763.2/PY) higher in residents than in non-residents. Compared to Chinese, excess costs were significantly higher in all other ethnic groups, with an increased $448.9/PY (95% CI: $425.4/PY-$471.4/PY) in Indian patients, $106.5/PY (95% CI: $88.2/PY-$124.8/PY) in Malay patients, and $267.6/PY ($236.2/PY-$299.0/PY) in patients of other ethnicities. On the other hand, socioeconomic status did not significantly influence excess costs. From 2012 to 2019, excess costs increased annually by an average of $75.2/PY (95% CI: $71.8/PY-$78.9/PY).

**Table 2:** Marginal effects of covariates on Per-PY excess costs

Patient Subgroup	Disease Category										
	ALL-CAUSE	ASTHMA	OCS-RELATED COMORBIDITIES	OTHER CIRCULATORY	OTHER DIGESTIVE	OTHER GENITOURINARY	OTHER MUSCULOSKELETAL	OTHER NERVOUS	OTHER METABOLIC	OTHER RESPIRATORY	ALL OTHERS
Age group, mean (95% CI)											
0–18 a	–	–	–	–	–	–	–	–	–	–	–
19-64	613.0∗∗∗ (593.6, 632.4)	39.8∗∗∗ (30.9, 48.7)	156.9∗∗∗ (151.4, 162.4)	124.1∗∗∗ (120.2, 128.0)	38.3∗∗∗ (34.6, 41.9)	29.7∗∗∗ (26.8, 32.6)	49.6∗∗∗ (46.2, 53.0)	3.9∗∗∗ (2.1, 5.8)	115.0∗∗∗ (111.8, 118.3)	−28.0∗∗∗ (−32.0, −24.1)	98.6∗∗∗ (91.9, 105.3)
65 or above	1701.5∗∗∗ (1669.8, 1733.1)	265.0∗∗∗ (250.3, 280.0)	472.9∗∗∗ (463.9, 481.9)	330.1∗∗∗ (323.8, 336.5)	70.2∗∗∗ (64.3, 76.1)	73.7∗∗∗ (68.9, 78.4)	79.6∗∗∗ (74.0, 85.2)	6.3∗∗∗ (3.3, 9.3)	267.6∗∗∗ (262.3, 272.9)	24.6∗∗∗ (18.1, 31.0)	198.6∗∗∗ (187.6, 209.6)
Gender, mean (95% CI)											
Female a	–	–	–	–	–	–	–	–	–	–	–
Male	−327.7∗∗∗ (−342.0, −313.4)	−107.1∗∗∗ (−115.3, −99.0)	−41.7∗∗∗ (−46.1, −37.2)	−14.6∗∗∗ (−17.7, −11.4)	−10.1∗∗∗ (−13.0, −7.2)	−27.4∗∗∗ (−29.7, −25.0)	−13.5∗∗∗ (−16.2, −10.7)	−1.8∗ (−3.2, −0.3)	−44.5∗∗∗ (−46.9, −42.0)	1.8 (−1.4, 4.9)	−101.2∗∗∗ (−106.6, −95.8)
Ethnicity, mean (95% CI)											
Chinese a	–	–	–	–	–	–	–	–	–	–	–
Indian	448.9∗∗∗ (426.4, 471.4)	145.0∗∗∗ (134.6, 155.2)	62.0∗∗∗ (55.6, 68.5)	37.0∗∗∗ (32.5, 41.6)	13.0∗∗∗ (8.8, 17.2)	24.3∗∗∗ (20.9, 27.7)	38.2∗∗∗ (34.2, 42.2)	8.4∗∗∗ (6.3, 10.5)	41.9∗∗∗ (38.1, 45.7)	32.6∗∗∗ (28.1, 37.2)	91.2∗∗∗ (83.4, 99.0)
Malay	106.5∗∗∗ (88.2, 124.8)	67.3∗∗∗ (59.0, 75.7)	0.2 (−5.0, 5.4)	−9.4∗∗∗ (−13.0, −5.7)	−12.7∗∗∗ (−16.1, −9.3)	9.9∗∗∗ (7.2, 12.7)	5.7∗∗∗ (2.5, 9.0)	2.7∗∗ (1.0, 4.4)	0.5 (−2.5, 3.6)	18.0∗∗∗ (14.3, 21.7)	45.5∗∗∗ (39.2, 51.9)
Others	267.6∗∗∗ (236.2, 299.0)	95.0∗∗∗ (80.5, 109.4)	19.3∗∗∗ (10.3, 28.3)	12.3∗∗∗ (6.0, 18.7)	13.1∗∗∗ (7.2, 19.0)	19.6∗∗∗ (14.9, 24.3)	23.0∗∗∗ (17.5, 28.6)	9.2∗∗∗ (6.2, 12.1)	10.2∗∗∗ (4.9, 15.5)	27.0∗∗∗ (20.6, 33.4)	70.6∗∗∗ (59.7, 81.5)
SES, mean (95% CI)											
Lower a	–	–	–	–	–	–	–	–	–	–	–
Middle	10.4 (−6.5, 27.3)	−0.7 (−8.3, 7.0)	0.8 (−4.0, 5.6)	−2.1 (−5.5, 1.3)	−0.8 (−3.9, 2.4)	2.5 (−0.1, 5.0)	4.6∗∗ (1.7, 7.6)	2.5∗∗ (0.9, 4.1)	3.2∗ (0.4, 6.1)	0.1 (−3.3, 3.5)	0.8 (−6.1, 6.6)
High	−12.6(-37.5, 12.2)	2.4 (−9.1, 13.8)	−4.9 (−12.0, 2.2)	−1.9 (−7.0, 3.1)	−3.3 (−7.9, 1.4)	0.0 (−3.7, 3.8)	7.2∗∗ (2.8, 11.6)	0.4 (−1.9, 2.8)	−1.5 (−5.7, 2.6)	−3.5 (−8.5, 1.6)	−6.5 (−15.2, 2.1)
Residency, mean (95% CI)											
Non-resident a	–	–	–	–	–	–	–	–	–	–	–
Resident	720.6∗∗∗ (678.0, 763.2)	108.4∗∗∗ (88.4, 128.3)	99.4∗∗∗ (87.3, 111.5)	84.8∗∗∗ (76.2, 73.3)	45.9∗∗∗ (37.9, 53.8)	27.8∗∗∗ (21.4, 34.2)	51.5∗∗∗ (44.0, 59.0)	18.0∗∗∗ (14.0, 22.0)	80.4∗∗∗ (73.3, 87.5)	76.8∗∗∗ (68.2, 85.5)	170.9∗∗∗ (156.2, 185.7)
Calendar year, mean (95% CI)	75.2∗∗∗ (71.6, 78.9)	20.5∗∗∗ (18.9, 22.1)	10.1∗∗∗ (9.1, 11.1)	12.4∗∗∗ (11.7, 13.2)	1.1∗∗ (0.4, 1.8)	2.1∗∗∗ (1.6, 2.7)	4.1∗∗∗ (3.4, 4.7)	0.4∗ (0.1, 0.7)	16.4∗∗∗ (15.8, 17.0)	6.6∗∗∗ (5.8, 7.3)	7.5∗∗∗ (6.2, 8.7)

**Abbreviations:** CI: confidence interval; OCS: oral corticosteroid; PY: patient-year; SES: socioeconomic status.

**Note**: All costs were measured in 2023 Singaporean dollars (SGD$1 = US$0.76 = ₤0.60 = €0.69). *p*∗<0.05, *p*∗∗<0.01, *p*∗∗∗<0.00.

a Reference group.

**Fig. 3:**
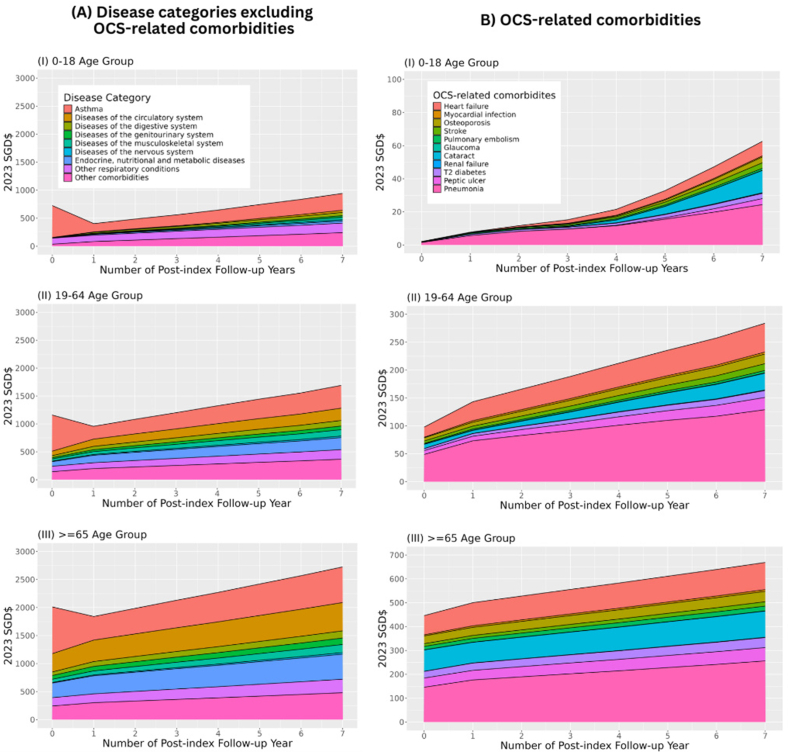
Trends of OCS-related Multimorbidity Costs in Asthma Patients by Age Group. Fig. 3 shows the trends of multimorbidity costs in asthma patients across follow-up years by age. All costs were measured in 2023 Singaporean dollars (SGD$1 = US$0.76 = ₤0.60 = €0.69). Abbreviations: OCS. oral corticosteroid; SGD: Singaporean dollar

Fig. 3 shows the trends of multimorbidity costs in asthma patients across follow-up years by age. Across all age groups, asthma-attributable costs peaked in the index year ($569.5/PY-$834.1/PY), sharply dropped after, and gradually rose back to nearly half by the eighth follow-up year ($300.6/PY–$635.6/PY). Over the disease course of asthma, in the paediatric group (aged 0–18 years), costs of non-asthma respiratory conditions were prominent and increasing ($107.9/PY-$170.0/PY), in addition to other specific cost groups including digestive ($2.5/PY-$57.0/PY) and musculoskeletal conditions ($1.5/PY-$44.1/PY). In the elderly group, costs of non-asthma respiratory conditions were 1.5 times higher as compared to the paediatric group ($148.6/PY-$240.9/PY), while OCS-related comorbidities ($435.5/PY-$654.3/PY) and circulatory diseases ($327.2/PY-$505.5/PY) emerged as major cost groups.

Costs of OCS-related comorbidities steadily increased up to 8 years since onset in the paediatric ($2.1/PY-$62.6/PY), adult ($97.9/PY-$283.9/PY), and elderly groups ($446.1/PY-$668.9/PY). Across all age groups, the larger cost components were pneumonia ($1.5/PY-$24.4/PY in children; $48.9/PY-$129.0/PY in adults; $145.9/PY-$256.4/PY in elderly) and cataract ($0.0/PY-$13.5/PY in children; $7.5/PY-$30.2PY in adults; $89.3/PY-$109.8/PY in elderly).

Table 3 displays results on prediction of high-cost asthma patients (cost comparisons between high and lower-cost patients presented in Supplemental Table 4). Asthma patients with the 10% highest per-PY costs of OCS-related comorbidities incurred a total of $3059.5/PY, which was $1596.2/PY higher than the rest, mainly driven by higher costs of primary care ($886.1/PY) and hospitalisations ($596.3/PY). These incremental costs were mostly associated with OCS-related comorbidities ($364.1/PY), other circulatory diseases ($257.5/PY), metabolic diseases ($219.9/PY), and asthma itself ($238.2/PY). LASSO-selected essential predictors of high OCS-related comorbidity costs were age (OR = 1.17 [95% CI: 1.16–1.17]), male sex (OR = 0.49 [95% CI: 0.48–0.5]), Indian ethnicity (OR = 2.94 [95% CI: 2.8–3.15]), duration of asthma (OR = 1.35 [95% CI: 1.33–1.36]), osteoporosis (OR = 1.1 [95% CI: 1.04–1.27]) and renal failure (OR = 1.13 [95% CI: 1.04–1.18]).

**Table 3:** Prediction of high-cost asthma patients

	10% highest Per-PY OCS-related comorbidity cost	≥2 Asthma Hospitalisations Per PY
**Per PY total costs, mean (95% CI)**		
Higher-burden subgroup^a^	3059.5 (3058.2, 3067.8)	2317.0 (2218.4, 2508.1)
Others	1463.2 (1461.7, 1463.4)	1588.3 (1586.0, 1587.9)
**Per PY excess costs by components, mean (95% CI)**		
Total	1596.2 (1586.0, 1601.6)	728.7 (447.3, 1063.0)
Hospitalisation	596.3 (594.3, 598.9)	282.4 (58.6, 341.8)
ED visit	29.7 (27.8, 30.3)	64.8 (30.6, 75.0)
Primary care visit	886.1 (881.8, 891.2)	333.8 (150.3, 352.2)
Specialist care visit	59.6 (59.5, 59.6)	25.0 (19.6, 29.2)
**Per PY excess costs by conditions, mean (95% CI)**		
Asthma	238.2 (237.2, 238.6)	90.4 (35.3, 118.3)
OCS-Related comorbidities	364.1 (363.6, 365.2)	146.2 (107.5,171.0)
Diseases of the circulatory system	257.5 (257.1, 258.4)	97.5 (41.6, 1102.5)
Diseases of the digestive system	58.4 (58.2, 58.7)	32.3 (21.6, 52.9)
Diseases of the genitourinary system	59.6 (59.3, 59.9)	30.9 (16.4, 26.2)
Diseases of the musculoskeletal system	73.9 (73.7, 74.3)	48.1 (42.6, 79.4)
Diseases of the nervous system	10.8 (10.7, 10.9)	8.7 (2.9, 16.9)
Endocrine, nutritional and metabolic diseases	219.9 (219.1, 220.5)	88.4 (55.9, 88.7)
Non-asthma respiratory conditions	70.8 (70.4, 71.2)	31.9 (2.8, 59.6)
Other comorbidities	215.7 (215.1, 216.9)	129.1 (126.4, 187.1)
**Essential predictors of high burdens**, OR (95% CI)	*(versus bottom 90%)*	*(versus <2 asthma hospitalisations)*
Age	1.17 (1.16, 1.17)	
Male gender	0.49 (0.48, 0.5)	0.81 (0.77, 0.87)
Ethnicity: Chinese	1 (1,1)	0.71 (0.69, 0.74)
Ethnicity: Indian	2.94 (2.8, 3.15)	1.03 (1.01, 1.06)
Ethnicity: Malay	0.9 (0.86, 0.99)	1 (1,1)
Ethnicity: Others	1 (1,1)	1 (1,1)
SES: Lower		
SES: Middle		
SES: Upper		
Duration of asthma	1.35 (1.33, 1.36)	
Heart failure		1.56 (1.17, 2.03)
Myocardial infection		
Osteoporosis	1.1 (1.04, 1.27)	
Stroke		
Pulmonary embolism		
Glaucoma		
Cataract		
Renal failure	1.13 (1.04, 1.18)	
Type 2 diabetes		
Peptic ulcer		
Pneumonia		2.93 (2.61, 3.23)
CRS: No CRS		
CRS: CRS without NP		
CRS: CRS with NP		
Allergic rhinitis		
Infectious diseases		1.1 (1.01, 1.17)
Tuberculosis		
Eczema		
Obesity		
Non-asthma respiratory conditions		
Diseases of the circulatory system		
Diseases of the digestive system		
Diseases of the genitourinary system		
Diseases of the musculoskeletal system		
Diseases of the nervous system		
Endocrine, nutritional, metabolic diseases		
Other comorbidities		

Note: The shaded cells represent predictors which were not selected by the LASSO algorithm. Allcosts were measured in 2023 Singaporean dollars (SGD$1 = US$0.76 = ₤0.60 = €0.69).

Abbreviations: CI: confidence interval; CRS: chronic rhinosinusitis; ED: emergency department; LASSO: Least Absolute Shrinkage and Selection Operator; NP: nasal polyps; OCS: oral corticosteroid; OR: odds ratio; PY: patient-year; SES: socioeconomic status.

a Higher-burden subgroup refers to: (in column 2) patients with the top 10% average OCS-related comorbidity costs and, (in column 3) patients who incurred 2 or more asthma hospitalisations over the study period.

Patients with recurrent asthma-related ED visits or hospitalisations incurred $2317.0/PY, which was $728.7/PY higher than the rest, mainly attributable to asthma ($90.0/PY), OCS-related comorbidities ($146.2/PY), circulatory diseases ($97.5/PY) and metabolic diseases ($88.4/PY). LASSO-selected predictors were male sex (OR = 0.81 [95% CI: 0.77–0.87]), Chinese ethnicity (OR = 0.71 [95% CI: 0.69–0.74]), Indian ethnicity (OR = 1.03 [95% CI: 1.01–1.06]), heart failure (OR = 1.56 [95% CI: 1.17–2.03]), pneumonia (OR = 2.93 [95% CI: 2.61–3.23]), and infectious diseases (OR = 1.1 [95% CI: 1.01–1.17]).

## Discussion

This study is by far the most comprehensive asthma multimorbidity cost study within a multi-ethnic Asian context. Based on national health administrative data from Singapore (2012–2019), we constructed an asthma cohort which consisted of 19,979 paediatric and 48,237 adult patients, primarily made up of Chinese (50%), Indian (14%) and Malay (27%) ethnicities. Our results revealed unique multimorbidity costing patterns in asthma patients, and high excess costs of OCS-related comorbidities. Paediatric asthma patients incurred $816/PY in total costs, while adult asthma patients incurred $1856/PY, which in combination corresponded to an average of $1611/PY. Of these costs, 31.7% were due to hospitalisations, 19.8% to ED visits and 48.5% to outpatient services including medications (44.6% primary care, 3.9% specialist care). Excess cost relative to non-asthma patients was $927/PY, which was significantly higher in females and the Indian ethnicity. Excess costs were mainly attributable to OCS-related comorbidities ($104/PY), other circulatory conditions ($113/PY), metabolic diseases ($116/PY), and non-asthma respiratory conditions ($107/PY). Our results also showed a steady increase in multimorbidity costs over the eight-year natural course of asthma, during which the per-PY costs of OCS-related comorbidities increased by $60 (30-fold) in the paediatric group and $200 (1.5–2.5-fold) in the adult and elderly groups.

Our estimates of asthma-attributable costs aligned with previous evidence^36^ and was similar to the Asian-Pacific regional average (USD320/PY).^37^ The estimated incremental all-cause direct medical costs ($927/PY) were largely consistent with global estimates from Canada (CAD1,059/PY)^4^ and Europe (€1555/PY).^38^ The main driver of excess costs was primary care costs (66%) which encompassed medication costs, aligning with cost composition in other countries.^4^^,^^39^ Comorbidity-attributable excess costs amounted to $524/PY, similar in scale with the Canadian asthma population (CAD689/PY);^4^ however, excess costs were driven by metabolic, circulatory, and non-asthma respiratory conditions, as opposed to psychiatric, digestive and nervous disorders in Canada.^4^ Contrary to non-increasing follow-up costs observed in the Western Caucasian population,^4^ over the time course of asthma, we found a steady rise in multimorbidity costs particularly in OCS-related comorbidities across all age groups. Alarmingly, this upward trend was also evident in children, whose costs would typically decline as asthma stabilises over time.^4^

Our findings highlighted the substantial incremental costs of OCS-related comorbidities in asthma patients, which amounted to 49% in incremental expense. Given previous evidence on the far-reaching effects of OCS use on overall health,^40^ the true excess costs could extend beyond the defined range of OCS-related comorbidities. As shown in our findings, the 10% highest per-PY OCS-related comorbidity costs were associated with elevated circulatory and metabolic excess costs ($258/PY and $220/PY) as high as asthma-attributable and OCS-related comorbidity-attributable costs themselves. These excess costs were predominantly driven by outpatient care and medications, possibly reflecting the additional effects of OCS prescriptions and the disparity in prevalence of Type 2 diabetes between the 2 cohorts. In proportion, our asthma cohort's OCS-related incremental costs were higher than Italy's (30%–45%),^9^ and a further breakdown revealed elevated costs of pneumonia and cataract – conditions with high underlying burden in Asia, surpassing those of Western populations and characterised by early disease onset.^41^^,^^42^ Of note, pneumonia is also a well-recognised complication of asthma itself, independent of OCS use.^43^ However, it has been shown that prolonged OCS exposure can increase susceptibility to infections, in particular pneumonia.^26^ In fact, OCS exposure and pneumonia risk has a dose-dependent relationship, with the risk in individuals using high doses of OCS being 2.3–3.5-fold that of non OCS-users.^44^ Therefore, in consistence with established categorisation, we included pneumonia as an OCS-related comorbidity. While pneumonia costs constitute 40%–50% of total OCS-related comorbidity costs across all age groups in our cohort, with its removal, OCS-related comorbidity costs would still be substantial, amounting to $85.2/PY-$106.5/PY. In addition, the consistent upward trend of OCS-related comorbidity costs observed in this study may reflect a growing OCS medication burden, given the possibility of increased OCS dependency over the course of asthma.^45^ This applies even to paediatric asthma, which calls for stronger preventive management of asthma.

In our Asian asthma population, we observed a unique multimorbidity pattern characterised by elevated excess burdens of circulatory diseases ($113/PY) and metabolic diseases ($116/PY), which were two-to three-fold of Western estimates (CAD54/PY and CAD41/PY). A possible explanation is the effects of OCS use, with previous evidence showing a dose-dependent association.^40^ Moreover, the Asian population had been associated with elevated burdens of circulatory and metabolic disorders.^46^ It is also likely that the high burden of OCS-related comorbidities among Indian patients was partially due to type 2 diabetes, given previous evidence suggesting its prominent association with Indian ethnicity in Singapore's severe asthma population.^15^ In our cohort, 23% of Indian patients had a diagnosis of type 2 diabetes, whereas the proportion was much lower in Chinese and Malay patients (12% and 14%). Factors contributing to the baseline predisposition include higher body mass index, inflammation, and insulin resistance, lower beta-cell function and high-density lipoprotein-cholesterol.^47^ Given Asia's unique genetic predisposition and significant ethnic variation, it is thus crucial to investigate the overall effects of OCS use on Asian asthmatics and their multimorbidity clusters. Contrary to Western multimorbidity patterns, our asthma cohort incurred low excess costs in psychiatric, nervous and digestive disorders, which were the top drivers of comorbidity-attributable excess costs in Western asthmatics. However, as our study data did not include outpatient visit records from private general practitioner (GP) clinics, it is likely that these comorbidity costs were not fully captured in our analysis. In Singapore, the government polyclinics and private GPs make up the primary care sector which is often the initial point of contact with patients. Of note, 80% of all primary care and 55% of all chronic care were provided by private GPs.^48^ Meanwhile, the deterring presence of stigma and a persistently low (∼30%) prevalence of mental-disorder help-seeking behaviour^49^ could have also led to underutilisation of psychiatric-related healthcare.

This study has several key strengths. First, by leveraging extensive population-based health administrative data, this study measured multimorbidity costs over long-term all-cause healthcare records and used robust propensity score matching to derive excess costs. Second, our study cohort consisted of a multi-ethnic Asian asthma population of predominantly Chinese, Indian and Malay patients, potentially allowing our findings to be generalised to >30% of the world population. However, several study limitations have to be acknowledged. First, due to a lack of prescription records, OCS exposure could not be directly measured in patients who were diagnosed with OCS-related comorbidities. While characterising healthcare costs by diagnosis categories enabled the identification of a set of OCS-related comorbidities and some estimation of OCS use, inability to distinguish patients with OCS exposure from those without could lead to overestimation of OCS-related comorbidity burden. Future studies should affirm the strengths of association between OCS use and OCS-related comorbidities in the Singapore asthma population. Second, we lacked health administrative data from private primary care settings, which may potentially affect the ethnic composition of the asthma cohort and also lead to under-representation of higher-SES, better managed asthma patients who had never incurred any asthma-related acute care, otherwise they would be already captured in our data. The direction of bias is multi-dimensional: on the one hand, costs of primary care and dispensed medications might be underestimated as private primary care attendees could experience greater disease complexity and incur higher costs;^48^ on the other hand, costs of acute care might be overestimated, as uncaptured private primary care patients never had any asthma-related acute care. Third, the inclusion of patients aged 0–5 years (10,720/68,216 asthma patients) may limit the generalisability of our findings pertaining to paediatric asthma. However, this revealed a predominance of early-diagnosed asthma which potentially reflects a common practice pattern in Asian settings. Fourth, unmeasured confounding during matching including the lack of smoking data could limit the comparability of the patient cohorts. Fifth, excess costs might be underestimated due to a lack of access to data on the general non-asthma population including individuals who used only private primary care or no care.

This study adopted the cost-of-Illness (COI) model to evaluate the economic impact of asthma multimorbidity from a healthcare system's perspective. Current cost estimates focused on monetary expenses directly associated with the diagnosis, treatment, care and management of asthma and comorbidities, which represented the forgone non-health market consumption redirected to healthcare and thus conveyed significant policy implications for optimising resource allocation.^50^ For instance, the substantial cost of hospitalisation underscores a critical need for shifting from a reactive urgent care model to emphasising preventive outpatient care. Also, the high and growing costs of certain comorbidities over the course of asthma, in particular OCS-related adverse events even among paediatric patients, highlighted the importance of integrated, streamlined care delivery and financing (e.g., value-based payment) across different levels of care. These findings may inform the next phase of strengthening the Healthier SG initiative. In addition, our findings can motivate the review of drug policies and drive strategies that mitigate the detriments of OCS-related treatment escalation, with new evidence showing that biologics initiation significantly reduces OCS use in individuals with severe asthma.^32^ Further, through a precision medicine approach, the identification of patient characteristics associated with differential outcomes in asthma patients can improve risk stratification in asthma management and strengthen preventive care. In particular, the elevated burden associated with the Indian ethnicity warrant further investigation to pinpoint causal factors leading to heightened risks and susceptibility, in order to facilitate timely and effective health interventions.

In conclusion, among asthma patients in Singapore primarily managed under public primary care, the burden of multimorbidity is high, with a substantial proportion of costs being attributable to OCS-related comorbidities. The broader effect of OCS use on overall health should be further investigated, while accounting for the unique multimorbidity clusters in the population. Policies should aim to reduce excess OCS use through optimised asthma care and integrated multimorbidity management.

## Abbreviations

CAD: Canadian dollar; CI: Confidence interval; ED: Emergency department; GP: General practitioner; HCU: Healthcare utilisation; ICD: International Classification of Diseases; LASSO: Least Absolute Shrinkage and Selection; OCS: Oral corticosteroid; OR: Odds ratio; PY: Patient-year; SES: Socioeconomic status; SD: Standard deviation; SGD: Singaporean dollar; SMD: Standardised mean difference; USD: US dollar.

## Author contributions

LHML contributed to the study design, data analysis and interpretation, and manuscript drafting and revision.

YRJ contributed to the design and conceptualisation of this study, the acquisition and interpretation of the data, and critical review of the manuscript draft.

SHC, MSK and JAA contributed to in funding acquisition, interpretation of the data, and critical review of the manuscript draft.

KBT contributed to in data acquisition, statistical supervision, interpretation of the data, and critical review of the manuscript draft.

DBP, MJT, MFL, PYT and ACAY contributed to data interpretation, and critical review of the manuscript draft.

WC was the supervisor of this work and contributed in funding acquisition, data acquisition, study design and conceptualisation, data interpretation, manuscript drafting and critical review. All authors provided their final approval of the manuscript version to be published, and agree to be accountable for the accuracy or integrity of all parts of the work.

## Ethics

This study complied with the Declaration of Helsinki. Ethics approval for exempted review was obtained from the Institutional Review Board (IRB) of National University of Singapore (NUS-IRB-2021-967).

## Disclosure of the use of Generative AI and AI-assisted technologies

Nothing to disclose.

## Role of funding source

This research is funded by the 10.13039/501100001352National University of Singapore Startup Grant and The Academic Respiratory Initiative for Pulmonary 10.13039/100018696Health (TARIPH), Singapore and supported by the 10.13039/501100001381National Research Foundation Singapore, Singapore under its Open Fund-Large Collaborative Grant (MOH-001636) and administered by the Singapore Ministry of Health's 10.13039/501100001349National Medical Research Council, Singapore. The funders had no role in the study design, data collection, analysis, or preparation of the manuscript.

## Declaration of competing interest

Sanjay H. Chotirmall reports grants from the Singapore Ministry of Education and Singapore Ministry of Health's National Medical Research Council, with payments made to the institution; consulting fees from Boehringer Ingelheim, CSL Behring, Pneumagen Ltd, Sanofi, GSK and Zaccha Pte Ltd; lecture fees from AstraZeneca, CSL Behring, Boehringer Ingelheim and Chiesi Farmaceutici; and participation on a data safety monitoring board or advisory board for Inovio Pharmaceuticals Inc. and Imam Abdulrahman Bin Faisal University. D.B. Price has advisory board membership with AstraZeneca, Boehringer Ingelheim, Chiesi, GlaxoSmithKline, Novartis, Viatris, and Teva Pharmaceuticals; consultancy agreements with AstraZeneca, Boehringer Ingelheim, Chiesi, GlaxoSmithKline, Novartis, Viatris, and Teva Pharmaceuticals; grants and unrestricted funding for investigator-initiated studies (conducted through OPRI) from AstraZeneca, Chiesi, Viatris, Novartis, Regeneron Pharmaceuticals, Sanofi Genzyme, and UK National Health Service; payment for lectures/speaking engagements from AstraZeneca, Boehringer Ingelheim, Chiesi, Cipla, Inside Practice, GlaxoSmithKline, Medscape, Viatris, Novartis, Regeneron Pharmaceuticals, Sanofi Genzyme, and Teva Pharmaceuticals; and payment for travel/accommodation/meeting expenses from AstraZeneca, Boehringer Ingelheim, Novartis, Medscape, and Teva Pharmaceuticals; owns 74% of the social enterprise Optimum Patient Care Ltd (Australia and UK) and 92.61% of OPRI Pte Ltd (Singapore); is peer reviewer for grant committees of the UK Efficacy and Mechanism Evaluation Programme, and Health Technology Assessment; and was an expert witness for GlaxoSmithKline. Dr Mariko Koh reports grant support from Astra-Zeneca, and honoraria for lectures and advisory board meetings paid to her hospital (Singapore General Hospital) from GlaxoSmithKline, Astra-Zeneca, Novartis, Sanofi, Boehringer Ingelheim and Roche outside the submitted work. John A. Abisheganaden reports grants from the Singapore Ministry of Health's National Medical Research Council, with payments made to the institution; Speaker or advisory board consulting fees from Pfizer, Astra Zeneca. Pei Yee Tiew has received honoraria for lecturers and advisory board meetings paid to her hospital from Sanofi and AstraZeneca outside the submitted work. Ming-Ju Tsai reports research grant for basic research of airway disease from the National Science and Technology Council (Taiwan); honoraria for lectures from GlaxoSmithKline, Astra-Zeneca, Novartis, Shionogi, Boehringer Ingelheim and Orient EuroPharma; and support for attending meetings from Pfizer and Shionogi. Mei Fong Liew has received lecture fees for GSK and Sanofi and serves on the advisory board for Astra Zeneca. Anthony Chau Ang Yii reports grants and honoraria from GlaxoSmithKline, Astrazeneca, and Sanofi. Wenjia Chen served as a delegate in the 2024 Early Career Investigators Meeting hosted by AstraZeneca and Global Respiratory Leadership Forum (GRLF) team and received research funding from Illumina.The rest of the authors declare no conflict of interest.
