# Supplementary material for: Economic burden of asthma multimorbidity in Singapore: Shadow costs of steroid use
Source: World Allergy Organ J. 2025 Nov 27;18(12):101146. doi: 10.1016/j.waojou.2025.101146 (PMC12701672; doi:10.1016/j.waojou.2025.101146)
Supplement: Multimedia component 1 [file mmc1.docx]

**Figure Legends**

**Supplemental Figure 1:** Schematic Diagram of Study Design. Abbreviations: COPD: Chronic obstructive pulmonary disease; ED: Emergency department; SOC: Specialist outpatient care.

**Supplemental Figure 2:** Patient Flowchart. Abbreviations: COPD: Chronic obstructive pulmonary disease; ED: Emergency department; SOC: Specialist outpatient care.

**Supplemental Figure 3:**  Descriptive Summary of Follow-up Healthcare Utilisation. Abbreviations: ED: Emergency department; OCS: Oral corticosteroid; PY: Patient-year.

**Supplemental Figure 1**: Schematic Diagram of Study Design

**
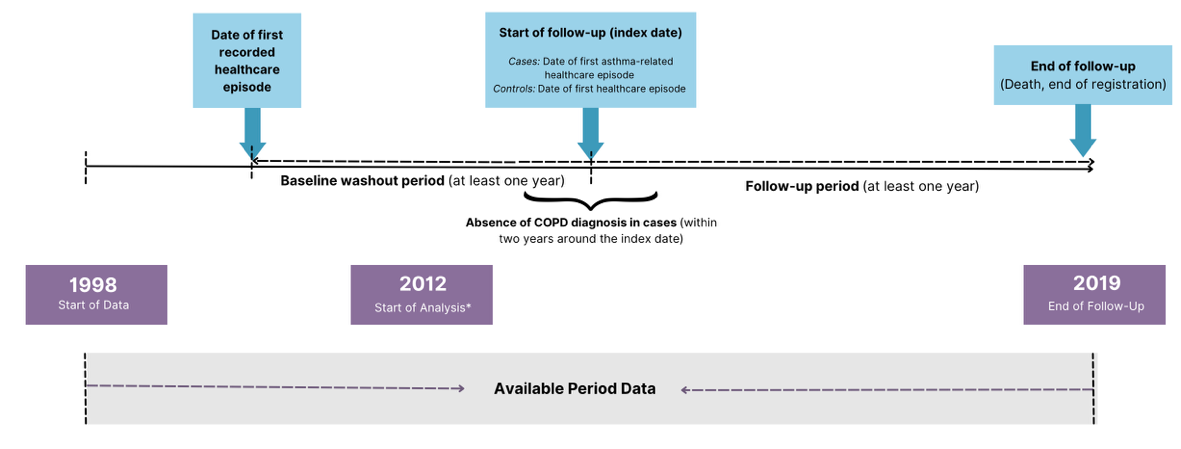
**

**Supplemental Figure 2**: Patient Flowchart

1. Asthma Patients


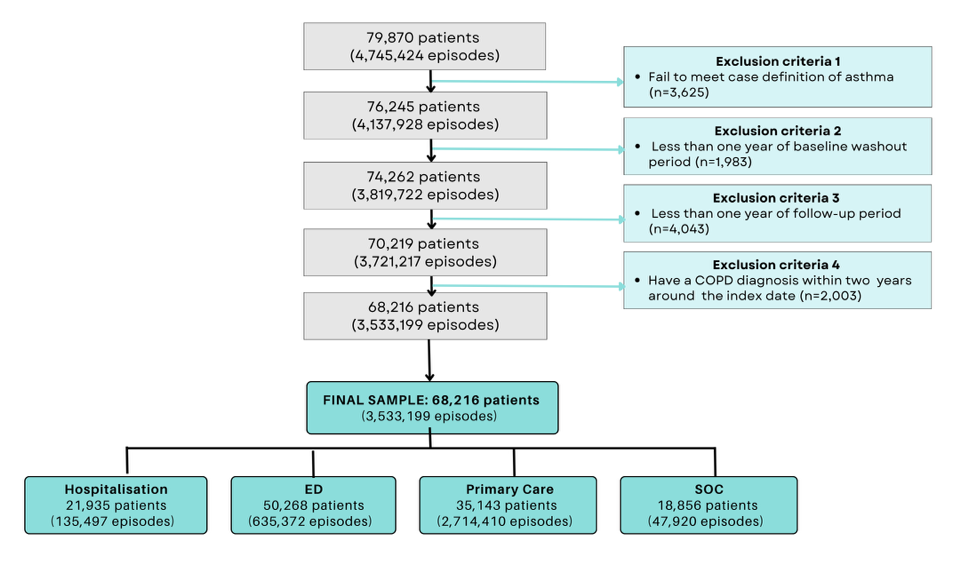


1. Non-asthma Patients


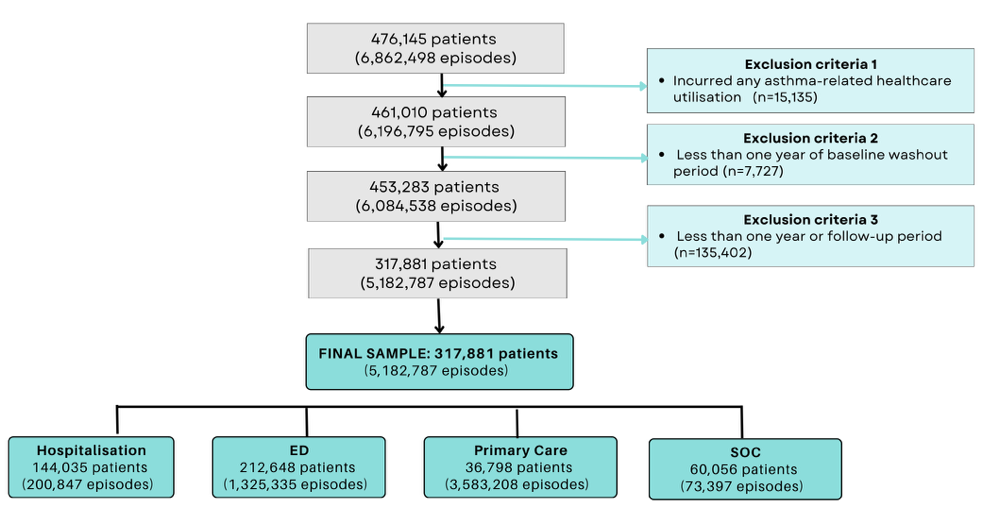


**Supplemental Figure 3:** Descriptive Summary of Follow-up Healthcare Utilisation


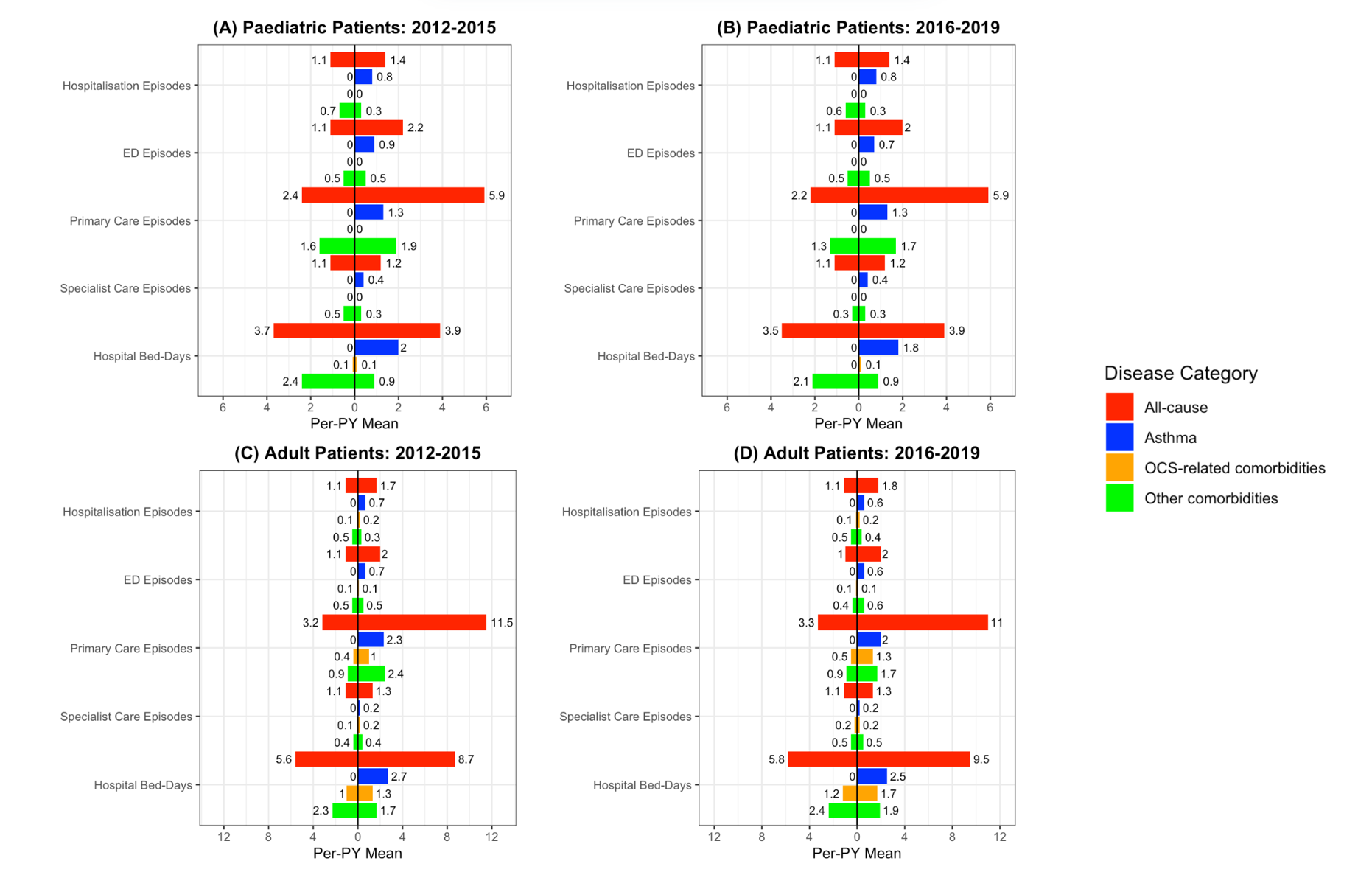


**Note:** The columns extending to the left of the y-axis represent the follow-up HCU volume of non-asthma patients. The columns extending to the right of the y-axis represent the follow-up HCU volume of asthma patients. Paediatric patients are those aged 0-18 years. Adult patients are those aged 19 years and above.
