# Supplementary material for: Economic burden of asthma multimorbidity in Singapore: Shadow costs of steroid use
Source: World Allergy Organ J. 2025 Nov 27;18(12):101146. doi: 10.1016/j.waojou.2025.101146 (PMC12701672; doi:10.1016/j.waojou.2025.101146)
Supplement: Multimedia component 2 [file mmc2.docx]

**Supplemental Table 1**: OCS-related Comorbidities

| Index | Comorbidity | ICD9 | ICD10 |
| --- | --- | --- | --- |
| 1 | Heart failure | 428 - Heart Failure | I50 - Heart Failure |
| 2 | Myocardial infarction | 410 - Acute myocardial infarction  412 - Old myocardial infarction | I21 - Acute myocardial infarction  I25.2 - Old myocardial infarction |
| 3 | Osteoporosis | 733.0 - Osteoporosis | M80 - Osteoporosis with pathological fracture  M81 - Osteoporosis without pathological fracture  M82 - Osteoporosis in diseases classified elsewhere |
| 4 | Stroke | 3623 - Retinal vascular occlusion  433 - Occlusion and stenosis of precerebral arteries  434 - Occlusion of cerebral arteries  436 - Acute, but ill-defined cerebrovascular disease  431 - Intracerebral hemorrhage  430 - Subarachnoid hemorrhage  435 - Transient cerebral ischemia | H34.1 - Central retina artery occlusion  I63 - Cerebral infarction  I64 - Stroke, not specified as haemorrhage or infarction  I61 - Intracerebral hemorrhage  I60 - Subarachnoid hemorrhage  G45 - Transient cerebral ischemic attacks and related syndromes |
| 5 | Pulmonary embolism | 415.1 - Pulmonary embolism | I26 - Pulmonary embolism |
| 6 | Glaucoma | 365 - Glaucoma | H40 - Glaucoma |
| 7 | Cataract | 366 - Cataract | H25 - Senile cataract  H26 - Other cataract |
| 8 | Renal failure | 584 - Acute renal failure  585 - Chronic renal failure  586 - Renal failure, unspecified | N17 - N19 - Renal failure |
| 9 | Depression ^a^ | 290.2 – Senile dementia, depressed or paranoid type  296.0 - Manic-depressive psychosis, manic type  296.1 - Manic-depressive psychosis, depressed type  296.2 - Manic-depressive psychosis, circular type but currently manic  296.3 - Manic-depressive psychosis, circular type but currently depressed  296.4 - Manic-depressive psychosis, circular type, mixed  296.5 - Manic-depressive psychosis, circular type, current condition not  296.6 - Manic-depressive psychosis, other and unspecified  298.0 - Depressive Type  300.4 - Neurotic depression  305.8 - Anti-depressants  309.0 - Brief depressive reaction  309.1 - Prolonged depressive reaction  311 - Depressive disorder, not elsewhere classified | F32 - Depressive episode  F33 - Recurrent depressive disorder |
| 10 | Anxiety ^a^ | 300.0 - Anxiety stress  313.0 - With anxiety and fearfulness | F40 - Phobic anxiety disorders  F41 - Oher anxiety disorders |
| 11 | T2 diabetes | 250 - Diabetes mellitus | E11 - Type 2 diabetes mellitus |
| 12 | Peptic ulcer | 533 - Peptic ulcer | K27 - Peptic ulcer, site unspecified, unspecified as acute or chronic, without hemorrhage or perforation |
| 13 | Pneumonia | 480 - Viral pneumonia  481 - Pneumococcal pneumonia [streptococcus pneumoniae pneumonia]  482 - Other bacterial pneumonia  483 - Pneumonia due to other specified organism  484 - Pneumonia in infectious diseases classified elsewhere  485- Bronchopneumonia organism unspecified  486-Pneumonia organism unspecified | J18. 9 |
| 14 | Obstructive sleep apnoea | 327.23-Obstructive sleep apnoea | G47.33 |

^a^ In line with TRUST requirements, diagnoses related to depression or anxiety were combined with a broader comorbidity category (Diseases of the Nervous System, see Table A2).

Abbreviations: ICD: International Classification of Diseases; OCS: oral corticosteroid.

**Supplemental Table 2**: Non-respiratory Major Comorbidity Categories

| Index | Disease Classification | ICD9 | ICD10 |
| --- | --- | --- | --- |
| 1 | Diseases of the circulatory system | 390-459 | I00-I99 |
| 2 | Diseases of the digestive system | 520-579 | K00-K93 |
| 3 | Diseases of the genitourinary system | 580-629 | N00-N99 |
| 4 | Diseases of the musculoskeletal system and connective tissue | 710-739 | M00-M99 |
| 5 | Diseases of the nervous system | 290-359 | F00-F99 & G00-G99 |
| 6 | Endocrine, nutritional and metabolic diseases | 240-279 | E00-E99 |
| 7 | Non-asthma respiratory conditions | 460–519 (excluding 493) | J00–J99 (excluding J45–J46) |

Abbreviations: ICD: International Classification of Diseases.

**Supplemental Table 3**: Annualised Total and Excess Costs by Component and Disease Category

| **Cost Component / Cohort** | **Disease Category** | | | | | | | | | | |
| --- | --- | --- | --- | --- | --- | --- | --- | --- | --- | --- | --- |
|  | **All-cause** | **Asthma** | **OCS-related** | **Other Circulatory** | **Other Digestive** | **Other Genitourinary** | **Other Musculoskeletal** | **Other Nervous** | **Other Metabolic** | **Other Respiratory** | **All Others** |
| Total    Asthma  Non-asthma    Excess | 1,610.9 (1,599.5-1,621.3)  525.8 (519.1-536.0)  927.2 (450.5-940.9) | 403.0 (400.3-406.5)  -  403 (85.1-732.6) | 213.0 (209.8-215.9)  78.91 (76.3-80.9)  104 (0-108.5) | 167.6 (165.6-170.0)  38.8 (37.5-41.5)  112.9 (0-115.0) | 77.9 (76.1-79.2)  55.5 (52.8-58.4)  13 (10.4-17.4) | 50.4 (48.9-51.8)  35.3 (33.9-37.7)  8 (0-10.8) | 80.8 (79.1-82.3)  35.3 (34.1-37.9)  32 (16.5-34.7) | 22.1 (21.4-22.8)  13.1 (11.7-14.5)  7 (5.0-8.3) | 158.9 (157.6-160.3)  25.5 (23.8-26.8)  116.4 (29.4-119.7) | 155.9 (154.2-158.0)  41.5 (39.6-43.1)  107.4 (96.0-109.5) | 281.1 (278.0-283.7)  210.0 (205.6-213.4)  53 (49.7-58.9) |
| Hospitalisation    Asthma  Non-asthma    Excess | 510.2 (503.4-516.5)  346.5 (341.7-355.1)  86.6 (0-93.6) | 173.8 (171.8-175.8)  -  158 (33.1-339.2) | 60.2 (58.4-61.8)  43.1 (40.9-45.8)  2 (0,4.5) | 25.0 (23.9-25.8)  20.8 (20.1-22.6)  0 (-2.2, 1.7) | 31.9 (31.7-33.3)  32.9 (31.6-34.7)  -8 (-10.6, -2.0) | 22.4 (21.4-23.2)  26.1 (23.1-26.1)  -8 (-9.7, 0) | 34.0 (33.2-34.9)  26.0 (24.6-28.2)  -2 (-4.3, 0.4) | 9.9 (9.4-10.7)  9.4 (9.1-10.6)  -1 (-2.4, 0) | 10.8 (10.1-11.1)  11.2 (10.3-12.4)  -3 (-4.6, -0.1) | 49.7 (48.1-50.8)  31.4 (29.5-32.4)  14 (12.1-16.7) | 102.7 (100.0-104.1)  154.5 (151.7-158.8)  -66 (-69, -39.2) |
| ED    Asthma  Non-asthma    Excess | 319.5 (316.5-322.2)  101.6 (99.8-103.3)  197.2 (177.4-200.9) | 77.7 (77.0-78.5)  -  73 (33.0-176.9) | 30.4 (29.1-31.1)  15.4 (14.6-16.7)  10 (0-11.9) | 10.9 (10.5-11.4)  6.4 (5.5-7.2)  3 (0.0-4.7) | 29.7 (28.7-30.2)  18.2 (17.4-19.5)  9 (7.5-11.3) | 13.2 (12.5-13.6)  8.4 (7.9-9.5)  4 (2.5-4.7) | 20.2 (19.5-20.9)  6.6 (6.1-7.0)  11 (3.7-12.3) | 8.6 (8.3-9.0)  3.0 (2.7-3.3)  5 (3.0-5.6) | 3.2 (3-3.3)  1.9 (1.7-2.2)  1 (0-1.3) | 38.9 (38.5-39.8)  6.9 (6.5-7.4)  32 (25.4-32.7) | 88.7 (87.4-89.6)  37.6 (36.4-39.2)  44 (42.2-45.7) |
| Primary Care    Asthma  Non-asthma    Excess | 717.8 (716.7-723.5)  51.9 (51.3-53.1)  613.4 (199.5-621.9) | 138.4 (137.2-139.3)  -  148 (0-275.1) | 103.9 (102.8-104.9)  10.0 (9.7-10.3)  83 (1.0-84.9) | 132.5 (131.5-133.5)  11.6 (11.3-11.9)  108 (0.8-110.0) | 11.0 (10.7-11.1)  1.1 (1-1.2)  9 (0-9.8) | 14.5 (14.1-15.0)  1.4 (1.3-1.4)  11 (0-12.6) | 27.0 (26.7-27.3)  2.5 (2.4-2.7)  22 (0-23.0) | 2.6 (2.6-2.8)  0.2 (0.2-0.3)  2 (0-2.4) | 144.7 (144.0-146.3)  12.0 (11.7-12.3)  119 (15.2-120.4) | 62.5 (62.0-62.9)  3.2 (3.1-3.3)  58 (19.1-58.2) | 85.2 (84.6-85.7)  10.5 (10.2-10.6)  69 (29.4-70.5) |
| Specialist Care    Asthma  Non-asthma    Excess | 63.4 (62.3-64.3)  30.4 (29.4-31.9)  30 (13.8-31.4) | 8.3 (8.0-8.5)  -  9 (0.4-18.2) | 21.3 (20.4-21.7)  11.4 (10.9-12.4)  8 (0.4-9.3) | 0.5 (0.5-0.6)  0.4 (0.4-0.5)  0 (0.1-0.2) | 6.4 (6.1-6.5)  3.8 (3.7-4.1)  3 (2.1-3.0) | 1.6 (1.4-1.7)  1.0 (1-1.1)  1 (0.3-0.7) | 1.3 (1.1-1.3)  0.7 (0.6-0.9)  0 (0.0-0.6) | 1.2 (1.1-1.3)  0.7 (0.5-0.7)  1 (0.1-0.7) | 1.1 (1.0-1.3)  1.0 (0.9-1.2)  0 (-0.1, 0.4) | 5.1 (4.9-5.2)  1.9 (1.7-2.1)  3 (2.1-3.4) | 17.7 (17.1-18.2)  10.3 (9.9-10.8)  7 (3.7-7.4) |

**Note**: Costs were presented in mean (95% CI confidence interval). All costs were measured in 2023 Singaporean dollars (SGD$1=US$0.76=₤0.60=€0.69).

**Abbreviations:** ED: emergency department; OCS: oral corticosteroid; SGD: Singaporean dollar.

**Supplemental Table 4**: Predicted Total Costs of High and Lower-Cost Asthma Patients

| **Cost Component** | **Disease Category** | | | | | | | | | | |
| --- | --- | --- | --- | --- | --- | --- | --- | --- | --- | --- | --- |
|  | **All-cause** | **Asthma** | **OCS-related** | **Other Circulatory** | **Other Digestive** | **Other Genitourinary** | **Other Musculoskeletal** | **Other Nervous** | **Other Metabolic** | **Other Respiratory** | **All Others** |
| By OCS-related comorbidity costs    Top 10%  Lower 90%    Excess | 3,059.5 (1,974.3-4,289.6)  1,463.2 (21.0-2,979.7)  1,596.2 (1,586.0-1,601.6) | 614.5 (360.0-908.9)  376.3 (98.1-741.6)  238.2 (237.2-238.6) | 548.6 (305.6-801.8)  184.5 (0-499.5)  364.1 (363.6-365.2) | 404.9 (207.5-580.5)  147.4 (0-379.6)  257.5 (257.1-258.4) | 131.7 (71.4-193.3)  73.3 (0-145.5)  58.4 (58.2-58.7) | 105.3 (59.7-153.3)  45.7 (0-109.0)  59.6 (59.3-59.9) | 148.9 (58.3-251.9)  75.0 (0-184.6)  73.9 (73.7-74.3) | 32.0 (9.7-56.5)  21.2 (1.8-44.7)  10.8 (10.7-10.9) | 361.6 (174.5-520.9)  141.7 (0-346.2)  219.9 (219.1-220.5) | 221.1 (132.1-313.9)  150.3 (58.9-255.8)  70.8 (70.4-71.2) | 491.0 (249.3-748.4)  275.3 (5.0-566.8)  215.7 (215.1-216.9) |
| Recurrent asthma hospitalisation/ED    Yes  No    Excess | 2,317.0 (941.3-3,531.4)  1,588.3 (34.8-3,425.5)  728.7 (447.3-1,063.0) | 485.4 (214.8-714.8)  395.0 (99.9-768.1)  90.0 (35.3-118.3) | 359.2 (109.2-560.8)  213.0 (0-625.1)  146.2 (107.5-171.0) | 265.1 (88.4-401.8)  167.6 (0-477.6)  97.5 (41.6-1102.5) | 110.1 (51.7-179.8)  77.9 (0-159.0)  32.3 (21.6-52.9) | 81.3 (27.9-119.1)  50.4 (0-122.2)  30.9 (16.4-26.2) | 128.9 (51.4-234.7)  80.8 (0-200.2)  48.1 (42.6-79.4) | 30.8 (16.6-54.7)  22.1 (2.2-47.0)  8.7 (2.9-16.9) | 247.3 (66.9-343.7)  158.9 (0-424.5)  88.4 (55.9-88.7) | 187.7 (99.8-307.9)  155.9 (59.9-269.6)  31.9 (2.8-59.6) | 421.2 (206.0-673.5)  292.2 (9.3-612.5)  129.1 (126.4-187.1) |

**Note**: Costs were presented in mean (95% CI confidence interval). All costs were measured in 2023 Singaporean dollars (SGD$1=US$0.76=₤0.60=€0.69).

**Abbreviations:** ED: emergency department; OCS: oral corticosteroid; SGD: Singaporean dollar.
